# Initial impacts of the COVID-19 pandemic on sexual and reproductive health service use and unmet need in Britain: findings from a quasi-representative survey (Natsal-COVID)

**DOI:** 10.1016/S2468-2667(21)00253-X

**Published:** 2022-01-04

**Authors:** Emily Dema, Jo Gibbs, Soazig Clifton, Andrew J Copas, Clare Tanton, Julie Riddell, Raquel Bosó Pérez, David Reid, Chris Bonell, Magnus Unemo, Catherine H Mercer, Kirstin R Mitchell, Pam Sonnenberg, Nigel Field

**Affiliations:** a Institute for Global Health, University College London, London, UK; b NatCen Social Research, London, UK; c Faculty of Public Health and Policy, London School of Hygiene & Tropical Medicine, London, UK; d MRC/CSO Social and Public Health Sciences Unit, University of Glasgow, Glasgow, UK; e Department of Laboratory Medicine, Örebro University, Örebro, Sweden

## Abstract

**Background:**

The COVID-19 pandemic has affected sexual and reproductive health (SRH) service use and unmet need, but the impact is unknown. We aimed to determine the proportion of participants reporting sexual risk behaviours, SRH service use and unmet need, and to assess remote sexually transmitted infection (STI) testing service use after the first national lockdown in Britain.

**Methods:**

We used data from the National Surveys of Sexual Attitudes and Lifestyles (Natsal)-COVID cross-sectional, quasi-representative web survey (Natsal-COVID Wave 1). Adults aged 18–59 years who resided in England, Scotland, or Wales completed the survey between July 29 and Aug 10, 2020, which included questions about the approximate 4-month period after announcement of the initial lockdown in Britain (March 23, 2020). Quota-based sampling and weighting were used to achieve a quasi-representative population sample. Participants aged 45–59 years were excluded from services analysis due to low rates of SRH service use. Among individuals aged 18–44 years, we estimated reported SRH service use and inability to access, and calculated age-adjusted odds ratios (aORs) among sexually experienced individuals (those reporting any sexual partner in their lifetime) and sexually active individuals (those reporting any sexual partner in the past year). Unweighted denominators and weighted estimates are presented hereafter.

**Findings:**

6654 individuals had complete interviews and were included in the analysis. Among 3758 participants aged 18–44 years, 82·0% reported being sexually experienced, and 73·7% reported being sexually active. 20·8% of sexually experienced participants aged 18–44 years reported using SRH services in the 4-month period. Overall, 9·7% of 3108 participants (9·5% of men; 9·9% of women) reported being unable to use a service they needed, although of the participants who reported trying but not being able to use a SRH service at least once, 76·4% of participants also reported an instance of successful use. 5·9% of 1221 sexually active men and 3·6% of 1560 sexually active women reported use of STI-related services and 14·8% of 1728 sexually experienced women reported use of contraceptive services, with SRH service use highest among individuals aged 18–24 years. Sexually active participants reporting condomless sex with new partners since lockdown were much more likely to report using STI-related services than those who did not report condomless sex (aOR 23·8 [95% CI 11·6–48·9]) for men, 10·5 [3·9–28·2] for women) and, among men, were also more likely to have an unsuccessful attempt at STI-service use (aOR 13·3 [5·3–32·9]). Among 106 individuals who reported using STI testing services, 64·4% accessed services remotely (telephone, video, or online). Among 2581 women aged 25–59 years, 2·4% reported cervical screening compared with an estimated 6% in a comparable 4-month period before the pandemic.

**Interpretation:**

Many people accessed SRH care during the initial lockdown; however, young people and those reporting sexual risk behaviours reported difficulties in accessing services and thus such services might need to address a backlog of need.

**Funding:**

Wellcome Trust, The Economic and Social Research Council, The National Institute for Health Research, Medical Research Council/Chief Scientist Office and Public Health Sciences Unit, and UCL Coronavirus Response Fund.

## Introduction

Sexual and reproductive health (SRH) care remains essential during the ongoing COVID-19 pandemic.^1^ However, SRH services, similar to the wider health-care system, were disrupted in Britain following implementation of a strict national lockdown announced on March 23, 2020, which mandated staying at home, except for essential shopping, medical care (in which access to SRH care was included), exercise, and some essential services.^2^ Despite the gradual easing of lockdown measures, some restrictions and physical distancing requirements remained throughout 2020.

**Research in context**

**Evidence before this study**

We searched PubMed and medRxiv from database inception to March 24, 2021, for research articles and preprints, using the search terms “coronavirus” or “COVID-19” or “pandemic” and “service use” or “service access” or “services” and “sexual health” or “STI” or “sexually transmitted infection” or “sexually transmitted disease” or “reproductive health” or “contraception” or “abortion.” Globally, few national studies on sexual and reproductive health (SRH) service use and sexual behaviour during the COVID-19 pandemic are available (limited to the USA and Australia), and no studies have been published from the UK. Most existing studies have relied on small online surveys, convenience samples, or participants recruited from clinical settings, which can result in biased estimates that are not representative of the general population. Previous studies have reported disruptions to SRH services due to the COVID-19 pandemic, and the continued need for service access among some high-risk populations.

**Added value of this study**

Natsal-COVID is the first large-scale, quasi-representative, national study of the impact of the COVID-19 pandemic on SRH and services in Britain. The study captured the impact of COVID-19 on SRH using rigorously designed questions. This web survey of 6654 participants aged 18–59 years found that 20·8% of participants aged 18–44 years reported using at least one SRH service in the 4-month period since the start of lockdown on March 23, 2020, in Britain, and 9·7% reported trying but being unable to use a service they needed. Use of contraceptive and sexually transmitted infection (STI)-related services was most commonly reported, and use was highest among young people (aged 18–24 years) and those reporting sexual risk behaviours such as condomless sex with a new partner. However, reporting trying but being unable to use services was also associated with risk behaviours, suggesting potential unmet need in the population. We also found that most participants reported using remote rather than face-to-face methods to access STI testing, including telephone, video, and online services.

**Implications of all the available evidence**

In response to the COVID-19 pandemic, SRH services in Britain rapidly adapted in the 4 months after the implementation of lockdown on March 23, 2020, to continue providing access for individuals in need, including a shift to remote modes of care. Our study provides population-level data on SRH service use and unmet need in Britain during this time. Despite pandemic-related restrictions, a minority of people reported sexual risk behaviours, highlighting continued need for SRH services. Contraception and STI services might need to address a backlog of need among high-risk groups, particularly young people (aged 18–24 years). There is also a cohort of women who might have missed cervical screening in the early months of the pandemic and might need additional follow-up. Our study provides context to interpret surveillance data for this period, and to inform likely pandemic impacts on SRH and services in other high-income countries.

The British Association for Sexual Health and HIV (BASHH) reported that many SRH services rapidly adjusted to facilitate access while reducing SARS-CoV-2 transmission risks. Measures included reducing face-to-face consultations, increasing remote interactions, suspending in-person clinics, and cancelling some appointments due to staff redeployment, illness, and self-isolation.^3^ Concerns about SARS-CoV-2 infection risk might also have decreased SRH service use. Subsequent delays to routine screening and diagnostic testing for sexually transmitted infections (STIs) and cervical screening might result in more diagnoses at a later stage, resulting in increased STI transmission and poorer cancer outcomes.^4^, ^5^, ^6^

The initial population-level impact of the COVID-19 pandemic on all forms of SRH service use and unmet need remains poorly understood. Most studies have relied on small convenience or clinic-based samples, which often lack representativeness and detailed information about sexual activity and risk behaviours.^3^, ^4^, ^7^, ^8^, ^9^ The National Surveys of Sexual Attitudes and Lifestyles (Natsal)-COVID study was conducted to understand how broad SRH and services in Britain were affected in the 4 months after the start of national lockdown in Britain in March, 2020. We hypothesised that some participants would have required SRH services during lockdown and would have experienced difficulties in access. We aimed to determine the proportion of participants reporting sexual risk behaviours, SRH service use and unmet need, and to assess remote STI testing service use.

## Methods

### Study design

Natsal-COVID Wave 1 was a cross-sectional, quasi-representative web survey of sexual health in Britain.^2^ Data were collected using a 10 min online questionnaire carried out by an online survey company (Ipsos MORI, London, UK). Existing members of Ipsos MORI's web-panel were contacted via email to participate in Natsal-COVID. Members sign up with Ipsos MORI and provide baseline information and regularly receive emails inviting them to take part in studies. Panellists receive small incentives to participate in Ipsos MORI surveys in the form of points, which can be redeemed for modest rewards and entry into sweepstake draws. The online panels are run with stringent recruitment and quality-control processes to ensure individuals can only join once, are not excessively sampled for surveys, and so remain engaged. Further recruitment details are described elsewhere.^2^ Eligible participants were aged 18–59 years who resided in England, Scotland, or Wales. Participants were asked about sexual behaviour and SRH service use in the approximate 4-month period after lockdown (appendix pp 1–3). To understand sociodemographic and behavioural risk factors, participants also provided data on sexual identity, ethnicity, education, general health and disability, mental health, alcohol consumption, and condomless sex. The full questionnaire^2^ is available online.

Ethical approval was obtained from the ethics committees of The University of Glasgow and London School of Hygiene & Tropical Medicine. Participants provided consent to participate via an online consent form before the start of the survey.

### Procedures

Data were collected between July 29 and Aug 10, 2020 (appendix p 4).^2^ Quotas were used to achieve a sample quasi-representative of the general population in Britain by age, gender, region (based on Office for National Statistics [ONS] 2019 mid-year estimates^10^), and social grade (based on ONS Census 2011 data^11^). The data were weighted to match the general population distribution by age, gender, sexual identity, region, social grade, and ethnicity. Full details of the weighting, and comparisons with probability surveys to illustrate representativeness, have been reported previously.^2^ Natsal-COVID was inclusive in its approach to gender, presenting data for all participants and separately for men (including transgender men) and women (including transgender women). Regarding gender, 24 participants identified in another way and were included in estimates presented for all. Further details of the Natsal-COVID methods are described elsewhere.^2^

### Statistical analysis

The target sample size was 6500 people comprising a core sample of 6000 aged 18–59 years and a boost sample of 500 people aged 18–29 years to improve the precision of estimates and enable more detailed analyses for this group who are at greatest risk of adverse SRH outcomes.^2^ We used complex survey analysis functions in Stata (version 16.1) to incorporate weighting and stratification.^2^ Unweighted denominators and weighted estimates are presented hereafter. We calculated population estimates for sexual risk behaviours (eg, new sexual partners and condomless sex with a new partner) and overall SRH service use and need in the approximate 4-month period from the start of lockdown (ie, at a time when meeting outside of households was prohibited). Men who have sex with men (MSM) were defined as men reporting any same-sex partnered sex in their lifetime. Participants aged 45–59 years were excluded from analysis of service use due to low rates of SRH service use among this age group. Participants were classified as having symptoms of depression or anxiety if they scored three or more on the two-item patient health questionnaire or two-item generalised anxiety disorder scales.^12^, ^13^

For overall SRH service use, estimates are presented by gender among sexually experienced participants (defined as individuals who reported any sexual partner in their lifetime) as the most relevant denominator. Reported STI-related service use (STI testing, STI follow-up care, and HIV testing) and unmet need (defined as participants reporting trying but being unable to use a service) are presented by gender and age among sexually active individuals (defined as reporting a sexual partner in the past year) aged 18–44 years. The sexually experienced category included participants defined as sexually active, and these denominators are presented in the appendix (p 6). Descriptive statistics for location and method of STI testing are presented for sexually active men and women aged 18–44 years. Data for participants reporting key risk behaviours (eg, condomless sex with a new partner) are also presented.

We calculated population estimates for reported contraception service use and unmet need by age among sexually experienced women aged 18–44 years. Reported use of abortion or pregnancy termination services among sexually experienced women aged 18–44 years, use of sexual assault or rape support services or helplines among all women aged 18–44 years, and cervical screening among all women aged 25–59 years are also presented. Surveillance data on abortion or pregnancy termination and cervical screening services were used to estimate uptake among women in the same age groups in a comparable 4-month period during normal circumstances.^14^

We used logistic regression to calculate age-adjusted odds ratios (aOR) to investigate how use of STI-related and contraception services and unmet need varied by sociodemographic and behavioural factors.

### Role of the funding source

The study funders had no role in study design, data collection, data analysis, data interpretation, or writing of the report.

## Results

**Figure 1:**
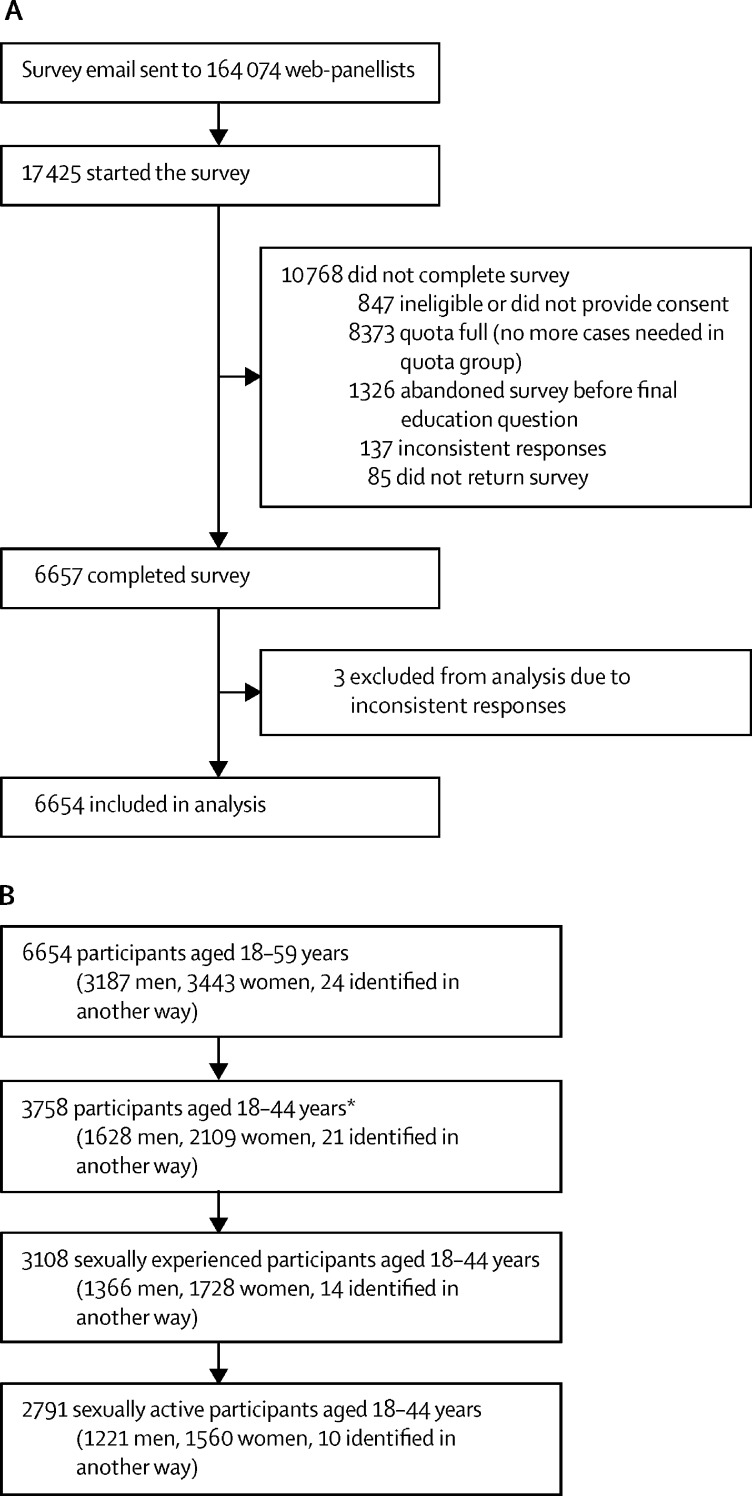
Recruitment process for Natsal-COVID (A) and sample selection for analysis of sexual and reproductive health service use (B) All numbers in this figure are unweighted. Natsal=National Surveys of Sexual Attitudes and Lifestyles. *654 participants did not provide information about previous sexual partners and were therefore excluded from these denominators.

6654 individuals had complete interviews and were included in the analysis (figure 1A). Among 3758 participants aged 18–44 years, 82·0% reported being sexually experienced, and 73·7% reported being sexually active (figure 1B). Characteristics of sexually experienced and sexually active participants are shown in table 1. Most sexually experienced men and women self-identified as heterosexual (95·5% [95% CI 94·7–96·1]) of sexually experienced men and 95·7% [95·0–96·3] of sexually experienced women), and 16·5% of sexually experienced men and 16·7% of sexually experienced women were in the youngest age group (18–24 years; table 1). Differences between unweighted and weighted denominators were largely due to the effects of weighting by sexual identity, and the young person's boost.

**Table 1:** Characteristics of sexually experienced and sexually active men and women aged 18–44 years in the 4 months following the start of a national lockdown in Britain (March 23, 2020)

	**Men**		**Women**	
	Sexually experienced, % (95% CI)	Sexually active, % (95% CI)	Sexually experienced, % (95% CI)	Sexually active, % (95% CI)
**Denominators**				
Unweighted	1366	1221	1728	1560
Weighted*	1457	1301	1449	1302
**Used at least one SRH service**				
No	84·7% (82·5–86·6)	83·6% (81·3–85·8)	73·9% (71·6–76·0)	72·6% (70·1–74·)
Yes	15·3% (13·4–17·5)	16·4% (14·2–18·7)	26·1% (24·0–28·4)	27·4% (25·1–29·9)
**Tried, but unable to use at least one SRH service**				
No	90·5% (88·7–92·1)	89·9% (87·9–91·6)	90·1% (88·4–91·5)	90·0% (88·3–91·5)
Yes	9·5% (7·9–11·3)	10·1% (8·4–12·1)	9·9% (8·5–11·6)	10·0% (8·5–11·7)
**Age, years**				
18–24	16·0% (14·0–18·3)	15·8% (13·7–18·2)	16·7% (15·1–18·5)	17·5% (15·8–19·5)
25–29	24·8% (22·4–27·3)	25·1% (22·6–27·8)	25·0% (23·0–27·0)	26·1% (24·0–28·3)
30–34	18·2% (16·1–20·5)	18·7% (16·4–21·1)	20·0% (18·0–22·3)	21·2% (19·0–23·7)
35–44	41·0% (38·2–43·8)	40·4% (37·4–43·3)	38·3% (35·7–40·9)	35·1% (32·5–37·8)
**Location**				
Urban	89·9% (88·0–91·6)	89·5% (87·5–91·3)	84·9% (82·8–86·7)	85·2% (83·0–87·1)
Rural	10·1% (8·4–12·0)	10·5% (8·7–12·5)	15·1% (13·3–17·2)	14·8% (12·9–17·0)
**Ethnicity**				
White†	83·5% (80·9–85·8)	84·0% (81·3–86·4)	83·5% (83·2–87·5)	85·8% (83·4–87·9)
Mixed, multiple, or other‡	3·8% (2·6–5·5)	3·8% (2·5–5·6)	3·4% (2·6–4·6)	3·4% (2·5–4·6)
Asian or Asian British§	9·5% (7·8–11·4)	9·3% (7·6–11·4)	7·9% (6·4–9·8)	7·7% (6·1–9·6)
Black or Black British¶	3·2% (2·2–4·8)	3·0% (1·9–4·6)	3·1% (2·2–4·5)	3·1% (2·1–4·6)
**Relationship status**				
Did not have partnered sex since lockdown	21·9% (19·6–24·3)	14·0% (12·0–16·3)	20·0% (18·0–22·1)	12·0% (10·4–13·8)
Partnered sex, not in a steady relationship	14·1% (12·2–16·2)	14·7% (12·7–17·0)	10·1% (8·7–11·8)	11·0% (9·4–12·7)
Partnered sex, in a steady non-cohabiting relationship	7·0% (5·7–8·6)	7·7% (6·2–9·4)	9·4% (8·0–10·9)	10·4% (8·9–12·1)
Partnered sex, in a steady cohabiting relationship	57·0% (54·2–59·8)	63·6% (60·6–66·4)	60·5% (58·0–63·0)	66·6% (64·1–69·1)
**Employment status**				
Employed	86·5% (84·4–88·4)	88·8% (86·7–90·7)	77·6% (75·4–79·6)	77·6% (75·3–79·8)
Unemployed	9·0% (7·4–10·8)	6·7% (5·3–8·4)	8·3% (7·0–9·8)	7·4% (6·1–8·9)
Full time parent, home maker	0·9% (0·5–1·6)	0·9% (0·5–1·6)	8·2% (6·9–9·8)	8·8% (7·4–10·6)
Student or pupil	3·6% (2·7–4·9)	3·6% (2·6–5·0)	5·9% (5·0–7·1)	6·1% (5·1–7·4)
**Self-reported sexual identity**				
Heterosexual	95·5% (94·7–96·1)	95·5% (94·7–96·2)	95·7% (95·0–96·3)	95·9% (95·3–96·5)
Gay or lesbian	3·0% (2·5–3·6)	2·9% (2·4–3·6)	1·1% (0·8–1·5)	1·1% (0·8–1·6)
Bisexual	1·3% (1·0–1·6)	1·3% (1·0–1·7)	2·6% (2·2–3·1)	2·5% (2·1–2·9)
Other	0·3% (0·1–0·7)	0·3% (0·1–0·6)	0·6% (0·4–1·0)	0·4% (0·2–0·8)
**Social grade**				
AB (higher and intermediate managerial, administrative, or professional occupations)	27·2% (24·9–29·8)	28·6% (26·0–31·3)	24·0% (21·9–26·2)	24·9% (2·7–27·3)
C1 (supervisory, clerical, and junior managerial, administrative, or professional occupations) or C2 (skilled manual occupations)	56·4% (53·6–59·2)	56·9% (53·9–59·9)	54·1% (51·5–56·6)	53·9% (51·2–56·6)
D (semi-skilled and unskilled manual occupations) or E (on state benefit, unemployed, and lowest grade occupations)	16·4% (14·3–18·7)	14·5% (12·4–16·9)	21·9% (19·9–24·1)	21·2% (19·1–23·4)
**Education**				
Degree	54·7% (51·9–57·5)	55·2% (52·2–58·2)	56·6% (54·1–59·1)	56·3% (53·6–58·9)
Below degree	42·1% (39·3–44·9)	41·8% (38·9–44·8)	40·9% (38·4–43·5)	41·1% (38·5–43·8)
No qualifications	3·2% (2·3–4·3)	3·0% (2·1–4·2)	2·5% (1·8–3·4)	2·6% (1·9–3·7)
**Alcohol consumption since lockdown**				
No change	50·6% (47·7–53·4)	50·9% (47·9–53·9)	59·7% (57·2–62·2)	59·2% (56·6–618)
Increased	28·6% (26·1–31·2)	28·9% (26·2–31·7)	22·0% (20·0–24·2)	22·8% (20·7–25·1)
Decreased	20·9% (18·6–23·3)	20·2% (17·9–22·7)	18·3% (16·4–20·4)	18·0% (16·0–20·1)
**Symptoms of depression (PHQ-2)**‖				
No	66·7% (63·9–69·3)	68·2% (65·4–71·0)	70·7% (68·3–73·0)	71·1% (68·6–73·5)
Yes	33·3% (30·7–36·1)	31·8% (29·0–34·6)	29·3% (27·0–31·7)	28·9% (26·5–31·4)
**Symptoms of anxiety (GAD-2)**‖				
No	69·2% (66·5–71·8)	69·7% (66·9–72·4)	67·8% (65·4–70·1)	67·4% (64·9–69·9)
Yes	30·8% (28·2–33·5)	30·3% (27·6–33·1)	32·2% (29·9–34·6)	32·6% (30·1–35·1)
New sexual partners since lockdown**				
None	92·0% (90·1–93·6)	90·9% (88·7–92·7)	97·6% (96·7–98·3)	97·3% (96·2–98·0)
At least one	8·0% (6·4–9·9)	9·1% (7·3–11·3)	2·4% (1·7–3·3)	2·7% (2·0–3·8)
Condom-less sex with a new partner since lockdown**				
No	93·8% (92·0–95·2)	92·9% (90·8–84·5)	98·2% (97·4–98·8)	98·0% (97·1–98·6)
Yes	6·2% (4·8–8·0)	7·1% (5·5–9·2)	1·8% (1·2–2·6)	2·0% (1·4–2·9)
**Previous same-sex experience in their lifetime**††				
No	91·1% (89·6–92·4)	91·7% (90·2–93·0)	92·2% (90·9–93·3)	92·5% (91·2–93·7)
Yes	8·9% (7·6–10·4)	8·3% (7·0–9·8)	7·8% (6·7–9·1)	7·4% (6·3–8·8)

SRH= sexual and reproductive health. PHQ-2=Patient health questionnaire two-item scale. GAD-2=Generalized anxiety disorder two-item scale.

* Participants aged 18–44 years; 14 participants who identified in another way were included in data presented for all participants, but excluded from the men and women categories; transgender men and transgender women were included in the men and women categories, respectively.

† Includes all individuals who identified as White English, Welsh, Scottish, Northern Irish, British, Irish, Gypsy or Irish Traveller, or from any other White background.

‡ Includes all individuals who identified as White and Black African, White and Black Caribbean, White and Asian or any other mixed or multiple ethnic background.

§ Includes all individuals who identified as Indian, Pakistani, Bangladeshi, Chinese, or from any other Asian background.

¶ Includes individuals who identified as African, Caribbean, or from any other Black background.

‖ Participants were classified as having symptoms of depression or anxiety if they scored ≥3 on the PHQ-2 or GAD-2 scales.

** Includes both partners of the opposite sex and same-sex partners.

†† Same-sex experience defined as oral, anal, or vaginal sex.

Regarding sexual risk behaviours, 86·0% of sexually active men and 88·0% of sexually active women reported partnered sex in the 4-month period following lockdown (data not shown). During this period, 9**·**1% (95% CI 7·3–11·3) of sexually active men and 2**·**7% (2·0–3·8) of sexually active women reported a new sexual partner (table 1), and this was more common among participants aged 18–24 years than those aged 35–44 years (OR 4·29 [95% CI 2·47–7·46], data not shown). Among 139 individuals who reported new sexual partners since lockdown, 80·6% (95% CI 72·2–86·9) reported condomless sex with a new partner during this period (data not shown). MSM (n=248) were more likely than other men to report new partners since lockdown (29·3% [95% CI 20·7–39·6] of MSM *vs* 7·3% [5·6–9·5] of men who did not report a previous same-sex experience, p=0·0050, data not shown). Of the 38 MSM who reported a new sexual partner since lockdown, 79·7% (62·6–90·2) reported condomless sex with a new partner (data not shown).

**Figure 2:**
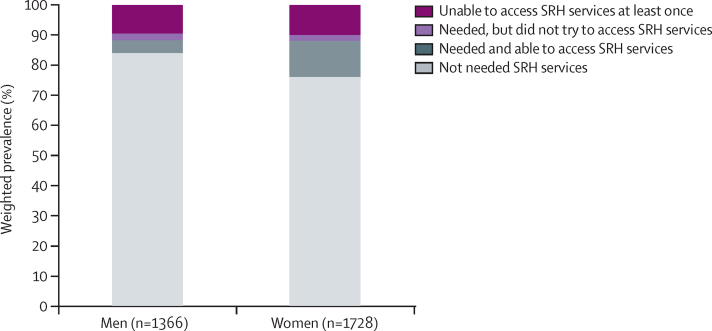
SRH service use among sexually experienced men and women aged 18–44 years in the 4 months following the start of a national lockdown in Britain (March 23, 2020) All percentages are weighted. Estimates for men and women included transgender men and transgender women, respectively. SRH=sexual and reproductive health.

The majority of sexually experienced participants aged 18–44 years reported not needing SRH services (84·0% [95% CI 81·8–86·0] of men; 76·0% [73·7–78·1] of women), whereas 8·3% (7·3–9·3; 4·4% [3·3–5·7] of men; 12·1% [10·5–13·8] of women) reported needing and being able to use services, 2·1% (1·6–2·7; 2·1% [1·4–3·2] of men; 2·0% [1·4–2·9] of women) reported needing services but not trying to access any, and 9·7% (8·6–10·8) of participants (9·5% [7·9–11·3] of men; 9·9% [8·5–11·6] of women) reported trying but not being able to use a SRH service at least once (figure 2). Of the 278 participants who reported trying but not being able to use a SRH service at least once, 76·4% also reported at least one instance of successful SRH service use, although this varied according to service type (data not shown). Overall, 20·8% (19·3–22·3) of sexually experienced participants aged 18–44 years reported using at least one SRH service since lockdown, and this was higher among women (26·1% [24·0–28·4]) than men (15·3% [13·4–17·5]; data not shown). The most frequently reported services were STI-related services and contraceptive services.

**Figure 3:**
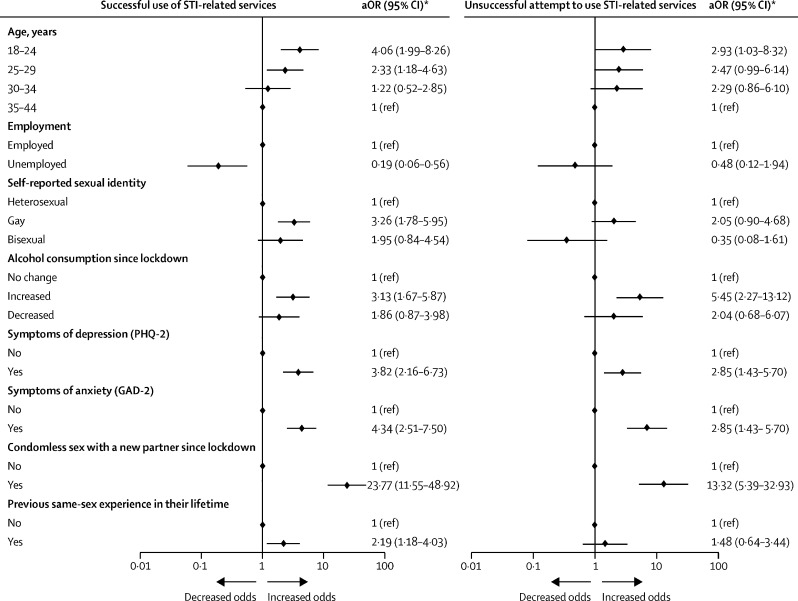
Forest plot of successful and unsuccessful attempts at STI-related service use among sexually active men aged 18–44 years (n=1197) in the 4 months following the start of a national lockdown in Britain (March 23, 2020) Weighted and unweighted denominators for each subgroup are presented in the appendix (pp 7–10). STI=sexually transmitted infection. aOR=adjusted odds ratio. PHQ-2=Patient health questionnaire two-item scale. GAD-2=Generalised anxiety disorder two-item scale. *All ORs are age-adjusted with the exception of those for the age categories, which are crude.

**Figure 4:**
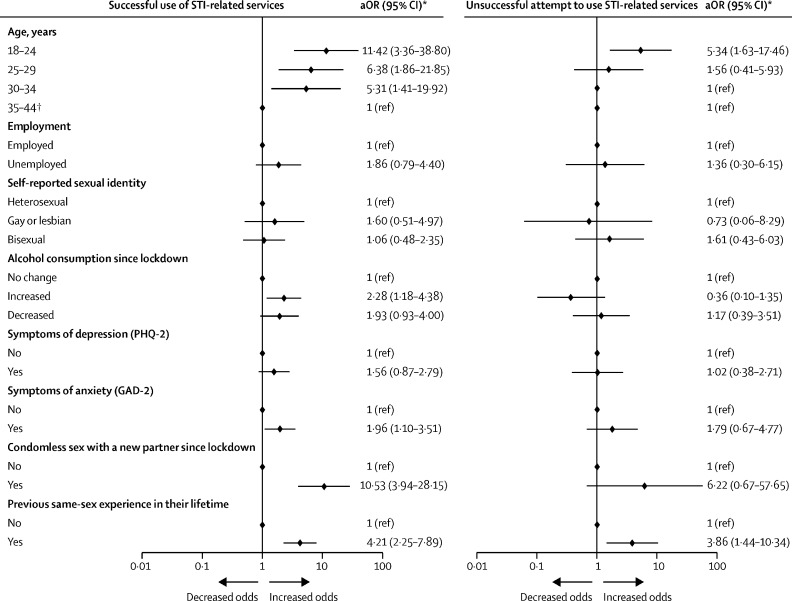
Forest plot of successful and unsuccessful attempts at STI-related service use among sexually active women aged 18–44 years (n=1548) in the 4 months following the start of a national lockdown in Britain (March 23, 2020) Weighted and unweighted denominators for each subgroup are presented in the appendix (pp 11–15). STI=sexually transmitted infection. aOR=adjusted odds ratio. PHQ-2=Patient health questionnaire two-item scale. GAD-2=Generalised anxiety disorder two-item scale. *All ORs are age-adjusted with the exception of those for the age categories, which are crude. †Age reference group for at least one failed attempt was 30–44 years.

Regarding STI-related services since the start of lockdown, men were more likely than women to report using these services (5·9% of men *vs* 3·6% of women) and use was highest in the youngest age groups (figures 3, 4). MSM and women reporting a previous same-sex experience (figure 4), were more likely to report using STI-related services than individuals who did not report a previous same-sex experience (aOR 2·19 [95% CI 1·18–4·03] for men; 4·21 [2·25–7·89] for women; figures 3, 4). Unemployed men were less likely than employed men to report using STI-related services (aOR 0·19 [0·06–0·56]; figure 3).

Several markers of poor health were associated with STI-related service use: after adjustment for age, men and women reporting increased alcohol consumption since the start of lockdown or symptoms of anxiety were more likely to report using STI-related services (aOR 3·13 [95% CI 1·67–5·87] for men and 2·28 [1·18–4·38] for women reporting increased alcohol consumption; 4·34 [2·51– 7·50] for men and 1·96 [1·10–3·51] for women reporting symptoms of anxiety), and these associations were stronger for men than women (figures 2, 3). Interactions between alcohol consumption and gender (p=0·69) and anxiety and gender (p=0·068) were not significant. Men, but not women, who reported depression symptoms were more likely to report STI-related service use (3·82 [2·16–6·73] for men; 1·56 [0·87–2·79] for women; figures 3, 4).

Strong positive associations were observed between reporting sexual behaviours associated with STI transmission and STI-related service use. For example, participants reporting condomless sex with a new partner since lockdown were much more likely to report using STI-related services than those who did not report condomless sex (aOR 23·8 [95% CI 11·6–48·9] for men, 10·5 [3·9–28·2] for women; figures 3, 4). However, many individuals who met guideline recommendations for STI testing did not report using services. For example, of 101 participants who reported condomless sex with a new partner since lockdown, 62·1% (95% CI 50·8–72·4) did not report using STI-related services in the same period (data not shown).

Of the 106 participants who reported STI testing, 49·4% had used a sexual health clinic, 32·6% had used a general practitioner, and 22·3% had used private or other services (table 2). 64·4% of 106 participants reported using remote methods of access rather than attending face-to-face, with telephone (31·5% of 106 participants) or online (33·4% of 106 participants) consultations more commonly reported than video consultations (table 2).

**Table 2:** Methods of accessing STI testing among sexually active participants aged 18–44 years in the 4 months following the start of a national lockdown in Britain (March 23, 2020)

	**Men, % (95% CI)**	**Women, % (95% CI)**	**Overall, % (95% CI)**
**Denominators**			
Unweighted	52	52	106
Weighted*	47	36	85
**Reported location of STI testing access**†			
Sexual health clinic	61·2% (45·3–75·1)	34·2% (21·8–49·2)	49·4% (38·6–60·3)
General practitioner	37·0% (23·1–53·5)	25·9% (15·0–40·9)	32·6% (23·1–43·7)
Private	7·6% (1·9–25·5)	5·4% (1·5–17·7)	6·4% (2·4–16·2)
Other‡	6·2% (2·3–15·6)	29·1% (17·5–44·1)	15·8% (9·9–24·4)
**Reported method of STI testing access**†			
Face-to-face	49·3% (33·8–64·9)	36·6% (23·8–51·6)	43·9% (33·4–55·0)
Remote	61·4% (45·1–75·4)	69·0% (54·6–80·5)	64·4% (53·4–74·0)
Telephone	31·6% (18·3–48·8)	33·2% (20·7–48·5)	31·5% (22·0–42·9)
Video	19·3% (9·4–35·6)	9·9% (3·7–24·1)	14·8% (8·3–25·2)
Online (and other)‡	28·4% (16·6–44·1)	39·0% (25·7–54·2)	33·4% (24·1–44·2)

STI=sexually transmitted infection.

* Participants aged 18–44 years who reported at least one sexual partner in the past year (ie, sexually active) and accessed STI testing services since lockdown.

† Participants were able to select more than one method or location; thus some percentages might exceed 100.

‡ Participants selected other; therefore it was not possible to determine which specific locations or methods were included in this response option.

A small minority of participants (2·2% [95% CI 1·7–2·9]) reported trying but being unable to use STI-related services (3·4% of men, 1·0% of women; appendix pp 7, 11). The patterns of association for unsuccessful attempts to use STI-related services were broadly similar to those for successful service use (figures 3, 4). Participants reporting sexual risk behaviours were significantly more likely to report unsuccessful attempts at accessing care (figures 2, 3). For example, 20·9% (95% CI 12·0–33·9) of 65 men who reported condomless sex with a new partner since lockdown also reported unsuccessful attempts to use STI-related services and were substantially more likely to do this than individuals who did not report condomless sex (aOR 13·3 [95% CI 5·3–32·9]; appendix p 10).

Among 2343 participants aged 18–44 years who reported partnered sex during lockdown, 17·9% (95% CI 15·5–20·6) of men and 6·2% (4·9–7·8) of women reported that they needed but were unable to access condoms since lockdown, and this was higher in the youngest age group (18–24 years) for men (33·9% [26·1–42·7]) and women (9·6% [6·4–14·2]; data not shown).

**Figure 5:**
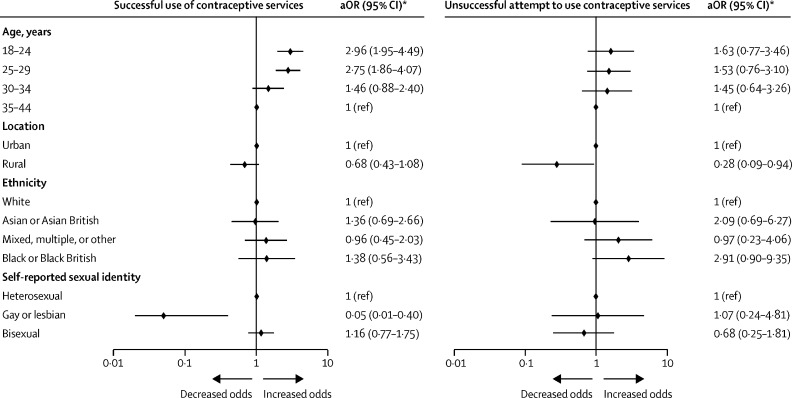
Forest plot of successful contraceptive service use and an unsuccessful attempt to use contraceptive services among sexually experienced women aged 18–44 years (n=1715) in the 4 months following the start of a national lockdown in Britain (March 23, 2020) Weighted and unweighted denominators for each subgroup are presented in the appendix (pp 15–18). aOR=adjusted odds ratio. *All ORs are age-adjusted with the exception of those for the age categories, which are crude.

Contraception was the main SRH service used by sexually experienced women; 14·8% (95% CI 13·1–16·6)reported using any contraceptive services since lockdown (appendix p 15). Younger participants (aged 18–24 years) were more likely to report use of contraceptive services than those aged 35–44 years (aOR 2·96 [95% CI 1·95–4·49]; figure 5; appendix p 15), but we observed no other associations with demographic characteristics. 4·0% of women reported trying but being unable to use contraceptive services since lockdown and this was less likely among women living in rural areas than among women living in urban areas (aOR 0·28 [0·09–0·94]; figure 4; appendix p 16). Among women using any form of contraception since lockdown, 6·0% (4·6–7·7) reported changing their contraception method in the same time period, and this was highest among those aged 18–24 years (9·2%) and 25–29 years (9·7%; data not shown).

15 sexually experienced women aged 18–44 years reported using abortion or termination of pregnancy services, giving a weighted estimate of 0·8% (0·5–1·4%), which was highest among those aged 18–24 years (1·9% [95% CI 0·9–4·1]; data not shown).

0·3% (95% CI 0·1–0·8) of women reported trying to but being unable to use abortion services, although this was also highest among the youngest age group (1·5% [0·5–4·3]; data not shown). We estimated approximately 0·6% of women aged 15–44 years accessed abortion services in a comparable 4-month period, based on data from 2019.^14^

13 women aged 18–44 years reported using sexual assault services since lockdown, giving a weighted population estimate of 0·6% (95% CI 0·3–1·1), which was highest (1·6% [0·8–3·5]) in the youngest age group (18–24 years; data not shown). Trying but being unable to use sexual assault services was reported by 0·3% (0·2–0·7) of women and was highest among the youngest women (1·1% [0·4–2·8]; data not shown).

Among 2581 women aged 25–59 years, 2·4% (1·8–3·1) reported using cervical screening services since lockdown, and 1·6% (1·2–2·2) reported trying but being unable to use services (data not shown). Of 46 women who were unable to use cervical screening services at least once, six (11·5%) also reported a successful attempt to use cervical screening services (data not shown), although it was unclear whether the failed attempt preceded the successful attempt. We estimated typical use of the cervical cancer screening programme among women aged 25–59 years to be around 6% for a comparable 4-month period before the pandemic.^15^

## Discussion

Our data indicate a continued need for and provision of SRH services during the first national COVID-19 lockdown in Britain. In the 4 months after the start of lockdown, 9·1% sexually active men and 2·7% sexually active women reported a new sexual partner, and most of these also reported condomless sex. 20·8% of sexually experienced participants reported using SRH services. 9·7% of participants reported an unsuccessful attempt to use a service they sought, but the majority (76·4%) of these individuals also reported successful use of one or more services. However, it is unclear whether the unsuccessful attempts preceded the successful attempts, nor whether the attempts were related to the same expressed need. Successful STI-related service use was higher in individuals who reported sexual risk behaviours, such as condomless sex with a new partner. Reporting an unsuccessful attempt to use STI-related services was also higher among individuals who reported sexual risk behaviours, highlighting potential unmet SRH needs in the population. Reflecting the rapid move of STI services to remote delivery,^3^, ^4^ most participants accessed these services remotely rather than in person. 31·5% of individuals reported using telephone services, 33·4% reported using online services, and 14·8% reported using video consultations. 17·9% of men struggled to access condoms in the 4 months from the start of lockdown, rising to 33·9% among men aged 18–24 years, suggesting that many individuals experienced difficulty accessing condoms, especially young people, which corroborates the findings of convenience sample research.^9^

Considering the wider focus on services beyond STI testing in this study, we also present data for SRH services such as abortion, sexual assault, and cervical cancer screening. The proportions of women who reported using abortion and sexual assault services since lockdown were small and should therefore be treated with caution, but indicate an important absolute number of women who needed these crucial and time-sensitive services during lockdown. For some of these services, we compared Natsal-COVID findings with expected uptake estimated using surveillance data. We estimated 0·6% of women aged 15–44 years accessed abortion services under normal circumstances in 2019, which is similar to findings from Natsal-COVID.^14^ Data from the Office for National Statistics illustrate that the number of abortions increased in England and Wales in 2020 compared with 2019, particularly among women older than 35 years, suggesting that abortion services have been resilient.^15^ At the start of the COVID-19 pandemic, health-care professionals in the UK adapted to provide early medical abortion care via postal delivery (eg, mifepristone or misoprostol), which is likely to have facilitated access to care during the initial phases of the pandemic.^16^ However, only 2·5% of women aged 25–59 years reported using cervical screening services during this 4-month period, which is lower than the estimated use of the cervical cancer screening programme among this age group for the same time period under normal circumstances (6%), suggesting a potential backlog of need.^17^

Natsal-COVID data support population-level data and four key trends suggested in other research. First, other research has shown sexual activity generally decreased during initial periods of lockdown; however, many individuals continued to report sexual risk behaviours, which was reflected in the Natsal-COVID data.^18^, ^19^, ^20^ This trend in behaviour was reflected by an overall decrease in SRH service use and STI reporting.^21^, ^22^, ^23^ However, symptomatic STI diagnoses in some clinical settings did not decrease, and a large proportion of individuals with new partners reported condomless sex,^7^, ^18^, ^19^, ^24^ suggesting a continued need for services. Second, young people (aged 18–24 years), who experience the greatest burden of STIs and unintended pregnancies, have been disproportionately impacted by service closures.^9^, ^24^, ^25^ For example, a web survey found that young people struggled to access free condoms and contraception, and many young people hesitated to use remote services.^9^ Although the risk of COVID-19 is lowest among young people,^26^ the impact of lockdown and SRH service disruptions seems highest in this group. Third, although demand for contraception largely continued, especially among individuals in cohabiting relationships, women have struggled to access contraception throughout lockdown, as evidenced by studies in the UK and globally.^3^, ^25^, ^27^, ^28^ Fourth, disruptions to cervical screening services have resulted in a backlog that will need to be addressed in subsequent months and years, as supported by studies from England and the USA.^5^, ^6^

The Natsal-COVID study has some important limitations. Research studies conducted during the COVID-19 pandemic required methodological adaptions to minimise SARS-CoV-2 transmission and adhere to government and ethical guidelines. The Natsal-COVID study was undertaken rapidly in response to the pandemic and benefited from a questionnaire design and approach developed by the team responsible for the decennial Natsal survey to obtain robust data on highly sensitive behaviours and experiences.^2^ Natsal-COVID included a large, national sample and used quota-based sampling and weighting to improve generalisability, and we aimed to achieve the best quality possible under circumstances where probability sampling and face-to-face interviews were not feasible.^29^, ^30^ Missing data were low for this study—eg, for most questions, fewer than 3% of data were missing. However, Natsal-COVID was not a probability sample, and therefore not fully representative of the general population.^2^ Findings are likely to be generalisable, but population estimates should be interpreted with appropriate caution. Baseline comparison data for service use and unmet need before lockdown were not available, and although we might compare data with estimates from the last decennial Natsal study,^31^ this study was undertaken between 2010 and 2012 and reported different timeframes (eg, 1 year). Due to small numbers of participants reporting new partners or condomless sex, we were unable to conduct multivariate analysis to determine factors associated with service use or failed attempts to access services among participants reporting risk behaviours. Similarly, due to small numbers of participants reporting use of STI testing services (n=106), we could not conduct further analysis on remote or in-person modes of service access. Compared with the decennial Natsal survey, which had a completion time of approximately 1 h, this web survey was shorter in length, restricting the amount of data we were able to collect. Further information on difficulty accessing SRH services, obtained through qualitative interviews, will be reported separately.

BASHH reported a shift from face-to-face to remote consultations, centralisation, and closure of some clinics, and an increase in individuals accessing online postal self-sampling in the first month of lockdown, reflecting changes already underway.^3^ Public Health England surveillance data are consistent, indicating an overall decrease in sexual health consultations, testing, and diagnoses between March and May, 2020, with a subsequent increase in diagnoses in June, 2020, when restrictions eased, although the number remained considerably lower than 2019 levels.^4^ The rapid shift to remote clinical consultations, testing, and management seems to have facilitated service access and aimed to prioritise in-person access for individuals and conditions most in need.^3^ Similarly, at a patient level, we expect that some individuals might have prioritised their immediate need for services over their preferred modality of service access. Remote STI testing also requires caution and regulation, since tests available from private providers might not always be clinically indicated or might have inadequate sensitivity.^32^

Our study could inform the design and development of future SRH services to meet backlogs and patient needs, including appropriate deployment of remote technologies in the recovery phase following the pandemic. We provide insights with implications for subsequent lockdown periods. Regardless of differences in how health systems are structured, financed, or commissioned in other high-income countries, these findings broadly indicate the likely impacts of the pandemic on SRH service use. These data also provide context to interpret surveillance data for this period, and identify services that might require support or strengthening. SRH services should prioritise engaging with young people,^23^ those reporting sexual risk behaviours, and women eligible for cervical screening to address the impact of the pandemic and prevent further service disruption and backlogs.

## Data sharing

An anonymised dataset is available from the UK Data Service.

## Declaration of interests

We declare no competing interests.
