# Supplementary material for: Initial impacts of the COVID-19 pandemic on sexual and reproductive health service use and unmet need in Britain: findings from a quasi-representative survey (Natsal-COVID)
Source: Lancet Public Health. 2022 Jan 4;7(1):e36–47. doi: 10.1016/S2468-2667(21)00253-X (PMC8730819; doi:10.1016/S2468-2667(21)00253-X)
Supplement: Supplementary appendix [file mmc1.pdf]

THE LANCET
Public Health

Supplementary appendix

This appendix formed part of the original submission and has been peer reviewed. We post it as supplied by the authors.

Supplement to: Dema E, Gibbs J, Clifton S, et al. Initial impacts of the COVID-19 pandemic on sexual and reproductive health service use and unmet need in Britain: findings from a quasi-representative survey (Natsal-COVID). *Lancet Public Health* 2022; **7:** e36–47.

Supplementary Material

Since the start of lockdown (23 March 2020), did you use any of the following sexual or reproductive health services? Please include phone, online or video appointments. Select all that apply.

- *None*
- *Contraception services/advice*
- *Fertility services/advice*
- *Maternity/antenatal services*
- *Abortion/Pregnancy termination services*
- *Cervical screening (smear test/pap test)*
- *STI (Sexually Transmitted Infection) testing*
- *STI follow-up care*
- *HIV testing*
- *Advice or counselling for sexual problems*
- *Relationship support services/advice*
- *Sexual assault/rape support services or helplines*
- *Other type of sexual or reproductive health service/advice*

Since the start of lockdown (23 March 2020), were there any sexual or reproductive health services which you tried to use but couldn't?

- *None – I have not needed to access services or advice*
- *None – I have been able to access the services I needed*
- *None – I would have liked to, but didn't try*
- *Contraception services/advice*
- *Fertility services/advice*
- *Maternity/antenatal services*
- *Abortion/Pregnancy termination services*
- *Cervical screening (smear test/pap test)*
- *STI (Sexually Transmitted Infection) testing*
- *STI follow-up care*
- *HIV testing*
- *Advice or counselling for sexual problems*
- *Relationship support services/advice*
- *Sexual assault/rape support services or helplines*
- *Other type of sexual or reproductive health service/advice*

Appendix A: Natsal COVID sexual and reproductive health (SRH) services questions

Participants who reported accessing sexually transmitted infection (STI) testing services were asked about their method and location of access. Response options were:

- *Face-to-face appointment*
    - *At a sexual health (GUM) clinic*
    - *At a GP*
    - *At a private (paid for) healthcare service*
    - *Somewhere else*
- *Telephone appointment*
    - *With a sexual health (GUM) clinic*
    - *With a GP*
    - *With a private (paid for) healthcare service*
    - *With another service*
- *Video appointment*
    - *With a sexual health (GUM) clinic*
    - *With a GP*
    - *With a private (paid for) healthcare service*
    - *With another service*
- *Other online service*
    - *With a sexual health (GUM) clinic*
    - *With a GP*
    - *With other NHS (free) service*
    - *With a private (paid for) healthcare service*
    - *With another service*
- *Other*
    - *Other type of appointment*

Appendix A: Natsal COVID sexual and reproductive health (SRH) services questions

Since the start of lockdown (23 March 2020), which of the following have you used to prevent pregnancy?

- *Does not apply – not had vaginal sex since lockdown*
- *Does not apply – currently pregnant*
- *Does not apply – trying to get pregnant*
- *Does not apply – cannot get pregnant*
- *No method used*
- *Condoms*
- *Oral contraceptive pill*
- *Hormonal IUS (e.g. Mirena, Jaydress, Kyleena, Levosert)*
- *Vaginal ring (e.g. NuvaRing)*
- *Contraceptive patch (e.g. EVRA)*
- *Injections*
- *Implants*
- *Copper coil/intra-uterine device (IUD)*
- *Emergency contraceptive pill/morning after pill*
- *Emergency copper coil/intra-uterine device (IUD)*
- *Safe period/calendar method/rhythm method*
- *Withdrawal (partner not ejaculating in vagina)*
- *Spermicides (foams/gels/sprays/pessaries)*
- *Other contraception*

Was there any time since lockdown when you needed to use any of the following, but didn't because you couldn't get hold of it?

- *Condoms*
- *Other contraception*
- *Medication for sexual problems (e.g. Viagra)*

Appendix A: Natsal COVID sexual and reproductive health (SRH) services questions

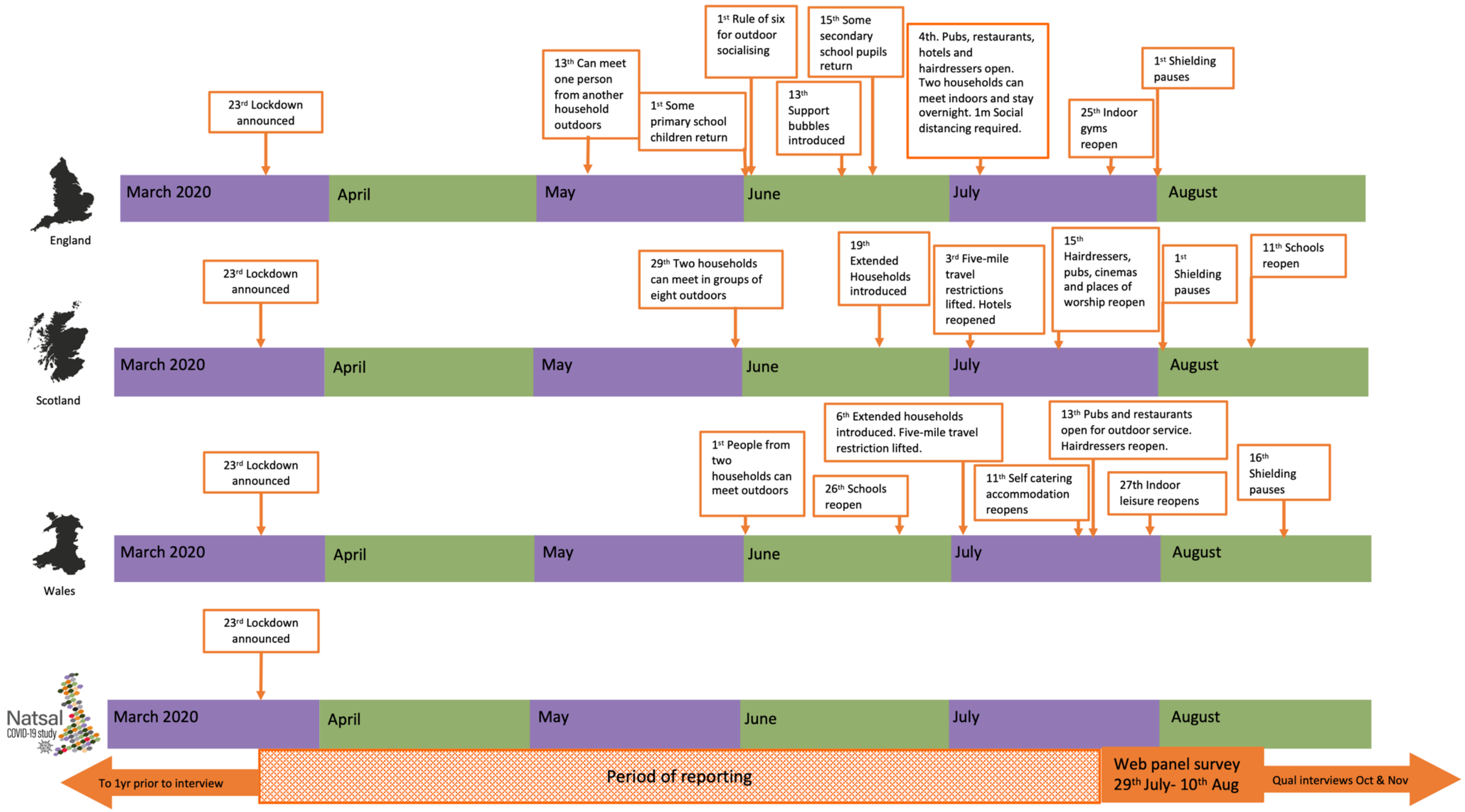

Appendix B: Timeline of Natsal-COVID study and COVID-19 restrictions on Britain.[2]

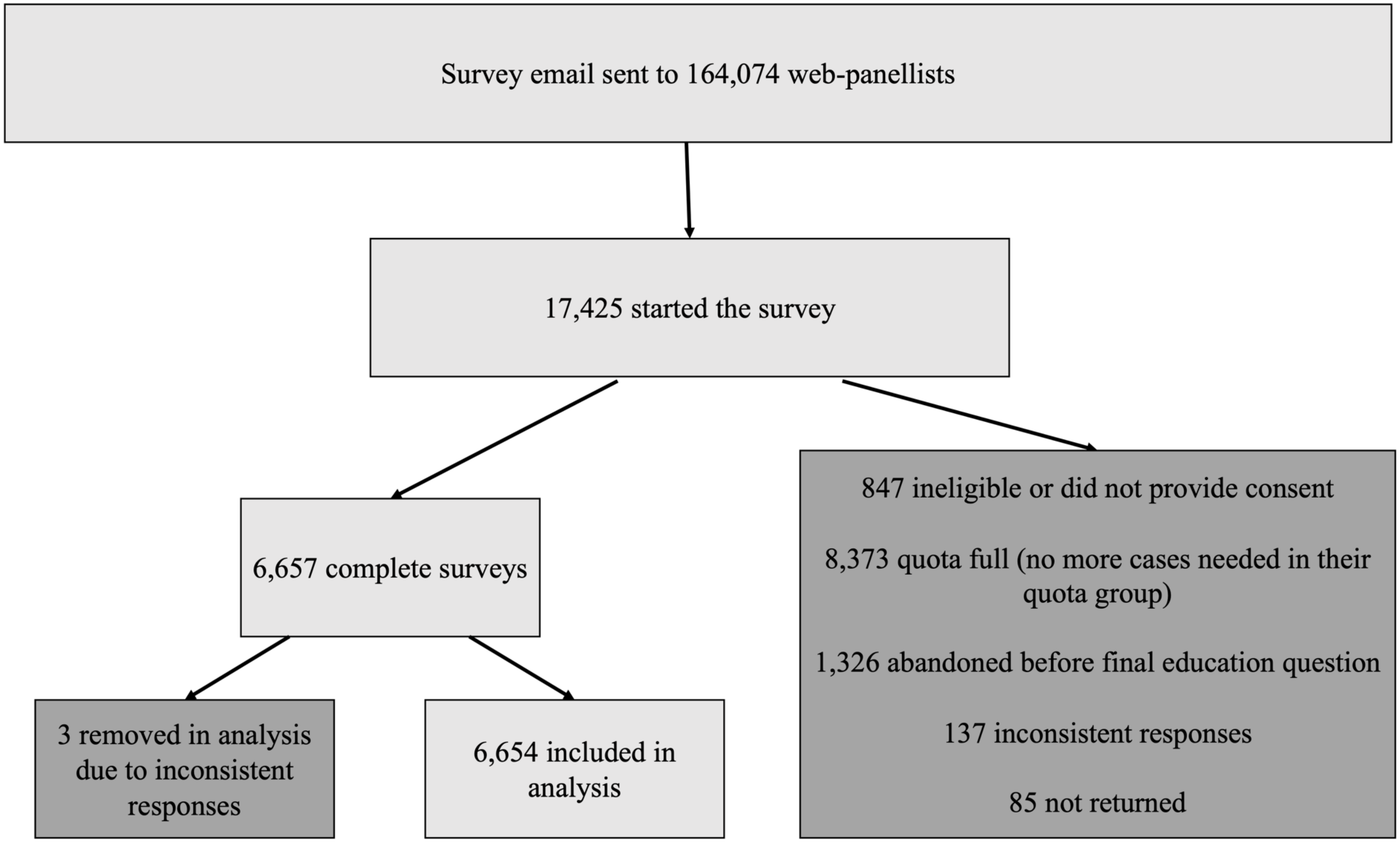

Appendix C: Recruitment process for Natsal-COVID. [2]

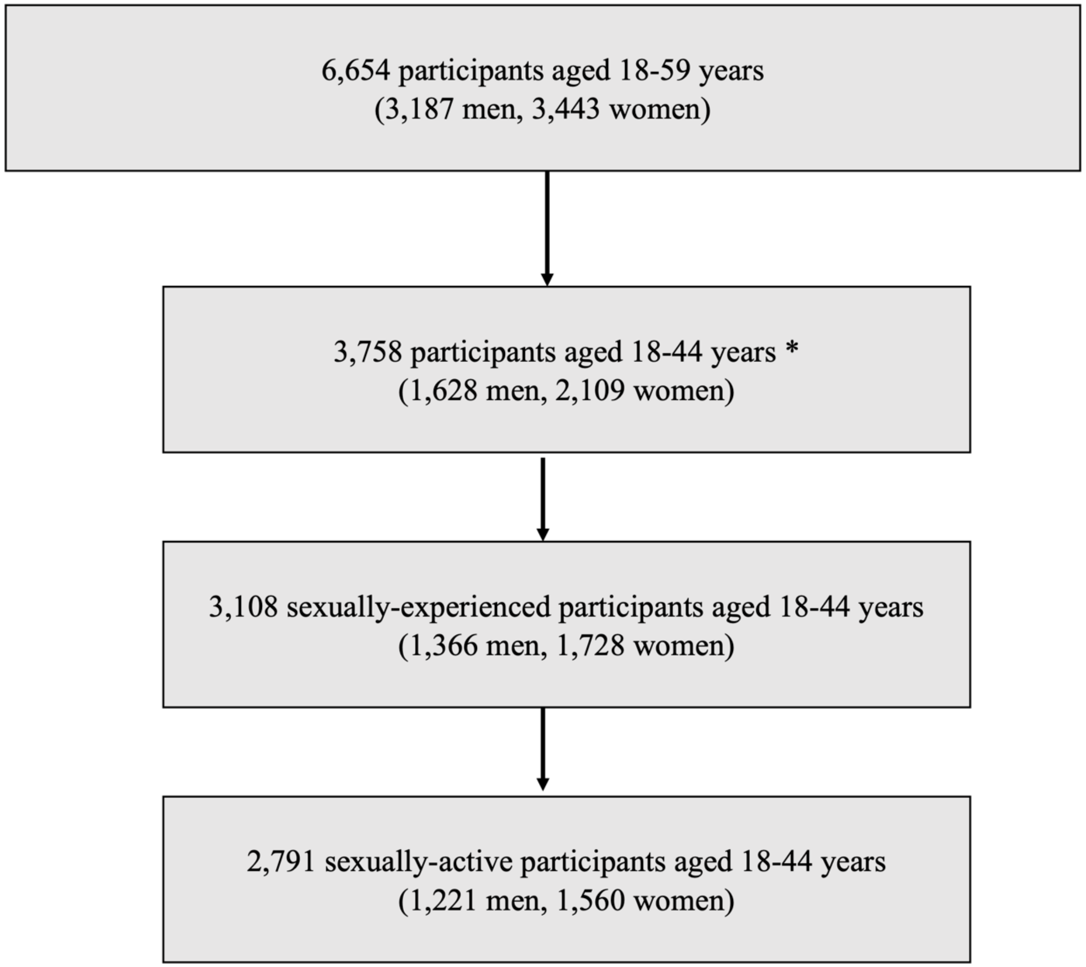

Appendix D: Sample selection for analysis of SRH service use.

*654 participants did not provide information about previous sexual partners, and are therefore excluded from these denominators

Appendix E: Variations in reporting successful sexually transmitted infection (STI)-related service use and an unsuccessful attempt to use STI-related services among sexually-active men aged 18-44 years (n=1197) in the four months following the start of a national lockdown in Britain (23/03/2020).

	Successful use				Unsuccessful attempt to use			
	% (95% CI)	OR (95% CI)	aOR (95% CI) ±	Denominator † (unweighted, weighted)	% (95% CI)	OR (95% CI)	aOR (95% CI) ±	Denominator † (unweighted, weighted)
**All sexually-active men aged 18-44 years**	5·9 (4·6 - 7·5)	··	··	1194, 1272	3·4 (2·4 - 4·7)	··	··	1197, 1276
**Age (years)**		**p=0·0006**				**p=0·15**		
18-24	12·3 (7·9 - 18·7)	4·06 (1·99 - 8·26)	··	188, 203	5·1 (2·4 - 10·4)	2·93 (1·03 - 8·32)	··	185, 200
25-29	7·5 (4·9 - 11·3)	2·33 (1·18 - 4·63)	··	299, 312	4·3 (2·5 - 7·5)	2·47 (0·99 - 6·14)	··	302, 317
30-34	4·0 (2·1 - 7·6)	1·22 (0·52 - 2·85)	··	218, 237	4·0 (2·1 - 7·7)	2·29 (0·86 - 6·10)	··	219, 238
35-44	3·3 (2·0 - 5·4)	1·00	··	489, 520	1·8 (0·9 - 3·6)	1·00	··	491, 521
**Location**		**p=0·76**	**p=0·60**			**p=0·90**	**p=0·99**	
Urban	6·2 (4·7 - 8·0)	1·00	1·00	951, 1012	3·2 (2·2 - 4·7)	1·00	1·00	954, 1017
Rural	5·4 (2·3 - 12·1)	0·87 (0·34 - 2·18)	0·78 (0·31 - 1·97)	116, 121	3·5 (1·2 - 9·5)	1·07 (0·34 - 3·35)	1·00 (0·32 - 3·16)	115, 120
**Ethnicity**		**p=0·023**	**p=0·053**			**p=0·038**	**p=0·070**	
White ***	5·0 (3·8 - 6·6)	1·00	1·00	1044, 1065	2·7 (1·8 - 3·9)	1·00	1·00	1042, 1062
Mixed/multiple/other ¶	4·6 (1·2 - 16·4)	0·91 (0·22 - 3·80)	0·75 (0·19 - 2·98)	36, 49 **	9·6 (3·6 - 23·4)	3·86 (1·25 - 11·89)	3·30 (1·10 - 9·94)	36, 49 **
Asian/ Asian British ¥	11·7 (6·4 - 20·7)	2·51 (1·21 - 5·22)	2·50 (1·19 - 5·26)	85, 111	6·7 (2·9 - 14·7)	2·61 (1·00 - 6·82)	2·56 (0·97 - 6·75)	88, 116
Black / Black British ¤	··	··	··	··	··	··	··	20, 38 *
**Relationship status**		**p=0·0060**	**p=0·051**			**p=0·015**	**p=0·067**	

Did not have partnered sex since lockdown	2·1 (0·7 - 6·0)	0·40 (0·13 - 1·23)	0·36 (0·11 - 1·16)	166, 178	1·2 (0·3 - 4·7)	0·43 (0·10 - 1·78)	0·39 (0·09 - 1·69)	167, 179
Partnered sex, not in a steady relationship	11·2 (7·1 - 17·2)	2·29 (1·24 - 4·23)	1·78 (0·91 - 3·48)	183, 177	7·7 (4·4 - 13·2)	2·84 (1·34 - 6·02)	2·28 (0·97 - 5·35)	184, 178
Partnered sex, in a steady, non-cohabiting relationship	9·0 (4·4 - 17·5)	1·81 (0·78 - 4·19)	1·31 (0·53 - 3·29)	89, 96	3·9 (1·2 - 11·5)	1·36 (0·39 - 4·81)	1·02 (0·26 - 3·95)	91, 99
Partnered sex, in a steady, cohabiting relationship	5·2 (3·7 - 7·2)	1·00	1·00	742, 806	2·9 (2·8 - 4·5)	1·00	1·00	743, 807
**Employment status**		**p=0·0068**	**p=0·0026**			**p=0·078**	**p=0.015**	
Employed	6·5 (5·1 - 8·3)	1·00	1·00	1057, 1129	3·6 (2·6 - 5·1)	1·00	1·00	1059, 1131
Unemployed	1·6 (0·6 - 4·3	0·23 (0·08 - 0·67)	0·19 (0·06 - 0·56)	81, 84	2·1 (0·6 - 7·8)	0·58 (0·14 - 2·36)	0·48 (0·12 - 1·94)	82, 86
Full time parent, home maker	··	··	··	12, 12 *	-	-	-	12, 12 *
Student/Pupil	0	0	0	44, 47 **	[0·4 (0·1 - 2·9)]	0·11 (0·01 - 0·82)	0·05 (0·01 - 0·43)	44, 47 **
**Sexual identity- self-reported**		**p=0·0002**	**p=0·0004**			**p=0·059**	**p=0·046**	
Heterosexual/straight	5·5 (4·2 - 7·1)	1·00	1·00	1006, 1210	3·3 (2·3 - 4·6)	1·00	1·00	1010, 1215
Gay	15·5 (9·9 - 23·5)	3·18 (1·76 - 5·74)	3·26 (1·78 - 5·95)	112, 37	6·5 (3·2 - 12·6)	2·05 (0·90 - 4·65)	2·05 (0·90 - 4·68)	113, 37
Bisexual	13·1 (6·6 - 24·3)	2·60 (1·16 - 5·86)	1·95 (0·84 - 4·54)	67, 17	1·6 (0·4 - 6·2)	0·47 (0·11 - 2·05)	0·35 (0·08 - 1·61)	66, 17
Other	··	··	··	5, 3 *	··	··	··	5, 3 *

**Social grade**		**p=0·15**	**p=0·14**			**p=0·31**	**p=0·30**	
AB Higher and intermediate managerial/administrative/professional occupation	6·5 (4·3 - 9·9)	1·00	1·00	375, 360	3·6 (2·1 - 6·3)	1·00	1·00	377, 362
C1 Supervisory, clerical and junior managerial/administrative/professional occupations/C2 Skilled manual occupations	6·5 (4·8 - 8·8)	0·99 (0·57 - 1·73)	0·98 (0·56 - 1·71)	658, 726	3·8 (2·5 - 5·7)	1·04 (0·50 - 2·13)	1·02 (0·49 - 2·10)	658, 726
D Semi-skilled and unskilled manual occupations/E On state benefit, unemployed and lowest grade occupations	2·4 (0·9 - 6·3)	0·35 (0·11 - 1·06)	0·34 (0·11 - 1·04)	161, 186	1·4 (0·4 - 4·7)	0·37 (0·09 - 1·48)	0·36 (0·09 - 1·43)	162, 187
**Education (3 categories)**		**p=0·72**	**p=0·74**			**p=0·91**	**p=0·89**	
Degree	5·4 (3·8 - 7·6)	1·00	1·00	662, 967	3·2 (2·0 - 5·0)	1·00	1·00	671, 708
Below degree	6·6 (4·6 - 9·3)	1·24 (0·73 - 2·09)	1·23 (0·73 - 2·10)	498, 537	3·7 (2·3 - 5·9)	1·15 (0·58 - 2·28)	1·15 (0·58 - 2·29)	491, 529
No qualifications	6·5 (1·6 - 22·7)	1·22 (0·28 - 5·31)	1·16 (0·24 - 5·55)	34, 38 **	2·8 (0·4 - 17·5)	0·87 (0·11 - 6·77)	0·83 (0·11 - 6·51)	35, 39 **
**Alcohol consumption since lockdown**		**p=0·0004**	**p=0·0016**			**p=0·0002**	**p=0·0003**	
No change	3·2 (2·0 - 5·1)	1·00	1·00	603, 645	1·3 (0·6 - 2·7)	1·00	1·00	601, 643
Increased	10·3 (7·3 - 14·3)	3·46 (1·87 - 6·41)	3·13 (1·67 - 5·87)	347, 363	7·3 (4·8 - 11·0)	5·96 (2·51 - 14·1)	5·45 (2·27 - 13·1)	350, 366
Decreased	6·8 (4·1 - 11·0)	2·20 (1·07 - 4·50)	1·86 (0·87 - 3·98)	237, 256	3·0 (1·5 - 6·1)	2·34 (0·82 - 6·67)	2·04 (0·68 - 6·07)	238, 257
**Symptoms of depression (PHQ-2) ******		**p<0·0001**	**p<0·0001**			**p=0·0010**	**p=0·0030**	

No	3·0 (1·9 - 4·5)	1·00	1·00	809, 861	1·9 (1·1 - 3·3)	1·00	1·00	813, 865
Yes	11·7 (8·6 - 15·6)	4·34 (2·48 - 7·59)	3·82 (2·16 - 6·73)	367, 390	6·0 (3·9 - 9·2)	3·26 (1·61 - 6·58)	2·85 (1·43 - 5·70)	367, 390
**Symptoms of anxiety (GAD-2) *****		**p<0·0001**	**p<0·0001**			**p<0·0001**	**p<0·0001**	
No	2·8 (1·9 - 4·3)	1·00	1·00	834, 886	1·1 (0·6 - 2·1)	1·00	1·00	835, 886
Yes	12·2 (8·9 - 16·5)	4·74 (2·75 - 8·19)	4·34 (2·51 - 7·50)	349, 374	8·1 (5·5 - 11·9)	7·68 (3·60 - 16·4)	7·10 (3·35 - 15·0)	352, 379
**New sexual partners since lockdown §**		**p<0·0001**	**p<0·0001**			**p<0·0001**	**p<0·0001**	
None	2·2 (1·3 - 3·5)	1·00	1·00	860, 924	1·4 (0·7 - 2·6)	1·00	1·00	867, 934
At least one	35·2 (24·9 - 47·1)	24·3 (12·1 - 48·8)	21·0 (10·2 - 43·2)	90, 89	19·3 (11·6 - 30·2)	16·8 (6·99 - 40·5)	14·7 (5·72 - 37·7)	88, 86
**Condom-less sex with a new partner since lockdown §**		**p<0·0001**	**p<0·0001**			**p<0·0001**	**p<0·0001**	
None	2·4 (1·6 - 3·8)	1·00	1·00	880, 939	1·7 (0·9 - 2·9)	1·00	1·00	887, 949
At least one	41·2 (28·8 - 54·8)	28·0 (13·8 - 57·1)	23·8 (11·5 - 48·9)	67, 69	20·9 (12·0 - 33·9)	15·7 (6·51 - 37·7)	13·3 (5·29 - 32·9)	65, 67
**Previous same-sex experience in their lifetime ‡**		**p=0·0029**	**p=0·012**			**p=0·24**	**p=0·36**	
No	5·4 (4·1 - 7·1)	1·00	1·00	987, 1168	3·2 (2·2 - 4·6)	1·00	1·00	989, 1171
Yes	12·0 (7·7 - 18·3)	2·40 (1·35 - 4·26)	2·19 (1·18 - 4·03)	207, 103	5·1 (2·5 - 10·2)	1·63 (0·72 - 3·72)	1·48 (0·64 - 3·44)	208, 105

CI=confidence intervals. OR=odds ratio. aOR=adjusted odds ratio. PHQ-2=Patient Health Questionnaire (2 item). GAD-2=Generalized anxiety disorder (2 item).

± Age-adjusted ORs, adjusting for age as a continuous variable
† Men aged 18-44 years who reported at least one sexual partner in the past year (i.e. sexually-active). Trans men are included in data for men. 27 and 24 sexually-active men aged 18-44 years responded “prefer not to say” to the questions about use and unsuccessful use of STI-related services, respectively. These individuals are excluded from the denominator.
* Unweighted denominator <30. Results not shown due to small denominator.
** Unweighted denominator <50. Results should be interpreted with caution due to small denominator.
*** White includes all those who identify as White English, Welsh, Scottish, Northern Irish, British, Irish, Gypsy or Irish Traveller, or from any other White background.
¶ Mixed ethnicity includes those who identify as White and Black African, White and Black Caribbean, White and Asian or any other mixed or multiple ethnic background.

¥ Asian includes those who identify as Indian, Pakistani, Bangladeshi, Chinese or from any other Asian background
¤ Black includes those who identify as African, Caribbean, or from any other Black background.
**** Participants were classified as having symptoms of depression or anxiety if they scored three or more on the patient health questionnaire two item (PHQ-2) or generalised anxiety disorder two item (GAD-2) scales
§ Includes both opposite-sex and same-sex partners
‡ Same-sex experience defined as oral/anal/vaginal sex

All percentages are weighted. These are row percentages which describe successful use/unsuccessful use of services within a specific subgroup.

Appendix F: Variations in reporting successful sexually transmitted infection (STI)-related service use and an unsuccessful attempt to use STI-related services among sexually-active women aged 18-44 years (n=1548) in the four months following the start of a national lockdown in Britain (23/03/2020).

	**Successful use**				**Unsuccessful attempt to use**			
	% (95% CI)	OR (95% CI)	aOR (95% CI) ±	Denominator † (unweighted, weighted)	% (95% CI)	OR (95% CI)	aOR (95% CI) ±	Denominator † (unweighted, weighted)
**All sexually-active women aged 18-44 years**	3·6 (2·7 - 4·7)	··	··	1548, 1292	1·0 (0·7 - 1·7)	··	··	1547, 1291
**Age (years)**		**p=0·0007**				**p<0·0001 *** **		
18-24	7·8 (5·2 - 11·5)	11·4 (3·36 - 38·8)	··	347, 224	2·9 (1·5 - 5·5)	5·34 (1·63 - 17·46)	··	345, 222
25-29	4·5 (2·9 - 6·9)	6·38 (1·86 - 21·9)	··	514, 337	0·9 (0·4 - 2·1)	1·56 (0·41 - 5·93)	··	517, 339
30-34	3·8 (2·0 - 7·1)	5·31 (1·41 - 19·9)	··	269, 274	1·5 (0·6 - 3·9)	1·00	··	270, 276
35-44	0·7 (0·2 - 2·3)	1·00	··	418, 457	0	1·00	··	415, 453
**Location**		**p=0·14**	**p=0·12**			**p=0·97**	**p=0·98**	
Urban	3·6 (2·6 - 5·0)	1·00	1·00	1131, 939	1·0 (0·6 - 1·7)	1·00	1·00	1131, 939
Rural	1·8 (0·7 - 4·4)	0·47 (0·17 - 1·28)	0·46 (0·17 - 1·22)	196, 164	1·0 (0·2 - 4·1)	1·03 (0·22 - 4·75)	0·98 (0·21 - 4·67)	195, 163

**Ethnicity**		**p=0·70**	**p=0·77**			**p=0·22**	**p=0·26**	
White ***	3·4 (2·6 - 4·5)	1·00	1·00	1377, 1095	1·2 (0·7 - 1·9)	1·00	1·00	1377, 1094
Mixed/multiple/other ¶	4·8 (1·2 - 17·2)	1·42 (0·33 - 6·04)	1·36 (0·30 - 6·12)	54, 44	0·0	0	0	54, 44
Asian/ Asian British ¥	2·8 (0·7 - 10··9)	0·82 (0·19 - 3·55)	0·89 (0·20 - 3·93)	71, 96	0·3 (0·0 - 2·4)	0·28 (0·04 - 2·15)	0·31 (0·04 - 2·41)	71, 97
Black / Black British ¤	··	··	··	29, 40 *	··	··	··	29, 40 *
**Relationship status**		**p<0·0001**	**p=0·0041**			**p=0·21**	**p=0·64**	
Did not have partnered sex since lockdown	6·0 (3·3 - 10·8)	3·39 (1·54 - 7·47)	2·56 (1·15 - 5·73)	190, 153	0·9 (0·3 - 2·8)	0·70 (0·17 - 2·88)	0·70 (0·17 - 2·88)	189, 152
Partnered sex, not in a steady relationship	8·1 (4·9 - 13·0)	4·65 (2·29 - 9·45)	3·30 (1·65 - 6·62)	183, 139	2·3 (0·9 - 5·7)	1·79 (0·54 - 5·90)	1·79 (0·54 - 5·90)	183, 138
Partnered sex, in a steady, non-cohabiting relationship	7·6 (4·2 – 13·4)	4·37 (1·99 - 9·58)	2·84 (1·22 - 6·62)	173, 133	2·0 (0·6 – 6·2)	1·35 (0·30 - 6·19)	1·35 (0·30 - 6·19)	173, 133
Partnered sex, in a steady, cohabiting relationship	1·9 (1·2 - 2·9)	1·00	1·00	977, 848	0·8 (0·4 - 1·5)	1·00	1·00	977, 848
**Employment status**		**p=0·033**	**p=0·26**			**p=0.94**	**p=0.58**	
Employed	3·3 (2·4 - 4·6)	1·00	1·00	1195, 1005	1·1 (0·6 - 1·8)	1·00	1·00	1193, 1003
Unemployed	6·2 (2·9 - 12·8)	1·90 (0·80 - 4·50)	1·86 (0·79 - 4·40)	119, 95	1·5 (0·4 - 5·8)	**1**·38 (0·30 - 6·33)	**1**·36 (0·30 - 6·15)	119, 96
Full time parent, home maker	0·7 (0·1 - 5·0)	0·21 (0·03 - 1·56)	0·27 (0·04 - 1·99)	117, 115	0·7 (0·1 - 5·0)	0·68 (0·09 - 5·25)	0·95 (0·12 - 7·64)	118, 115
Student/Pupil	7·7 (3·8 - 15·0)	2·40 (1·06 - 5·46)	0·97 (0·42 - 2·23)	117, 77	0·9 (0·1 - 5·9)	0·82 (0·11 - 6·33)	0·25 (0·03 - 1·94)	117, 77

**Sexual identity- self-reported**		**p=0·33**	**p=0·80**			**p=0·12**	**p=0·39**	
Heterosexual/straight	3·5 (2·6 - 4·6)	1·00	1·00	1342, 1232	1·0 (0·6 - 1·6)	1·00	1·00	1341, 1231
Gay or Lesbian	6·8 (2·4 - 17·4)	2·01 (0·66 - 6·07)	1·60 (0·51 - 4·97)	40, 15 **	1·2 (0·2 - 8·4)	1·27 (0·16 - 9·93)	0·73 (0·06 - 8·29)	40, 15 **
Bisexual	5·2 (2·6 - 10·1)	1·52 (0·70 - 3·30)	1·06 (0·48 - 2·35)	148, 32	2·3 (0·7 - 7·2)	2·42 (0·67 - 8·76)	1·61 (0·43 - 6·03)	147, 32
Other	··	··	··	10, 5 *	··	··	··	11, 6 *
**Social grade**		**p=0·84**	**p=0·75**			**p=0·47**	**p=0·44**	
AB Higher and intermediate managerial/administrative/professional occupations	3·1 (1·8 - 5·2)	1·00	1·00	406, 323	1·6 (0·7 - 3·3)	1·00	1·00	406, 322
C1 Supervisory, clerical and junior managerial/administrative/professional occupations/C2 Skilled manual occupations	3·8 (2·6 - 5·4)	1·22 (0·63 - 2·36)	1·29 (0·66 - 2·52)	807, 695	0·9 (0·5 - 1·9)	0·61 (0·22 - 1·70)	0·64 (0·23 - 1·83)	808, 697
D Semi-skilled and unskilled manual occupations/E On state benefit, unemployed and lowest grade occupations	3·7 (2·1 - 6·4)	1·20 (0·54 - 2·66)	1·12 (0·50 - 2·51)	335, 274	0·7 (0·2 - 2·4)	0·44 (0·10 - 1·95)	0·39 (0·09 - 1·77)	333, 272
**Education (3 categories)**		**p=0·069**	**p=0·046**			**p=0·27**	**p=0·20**	
Degree	4·2 (3·0 - 5·8)	1·00	1·00	863, 725	1·3 (0·7 - 2·3)	1·00	1·00	865, 727
Below degree	2·5 (1·5 - 4·0)	0·59 (0·32 - 1·07)	0·55 (0·30 - 1·01)	644, 533	0·7 (0·3 - 1·7)	0·56 (0·20 - 1·56)	0·50 (0·17 - 1·46)	642, 531
No qualifications	8·6 (2·8 - 23·8)	2·17 (0·62 - 7·56)	2·21 (0·60 - 8·15)	41, 34 **	0	0	0	40, 32 **
**Alcohol consumption since lockdown**		**p=0·011**	**p=0·036**			**p=0·19**	**p=0·23**	
No change	2·3 (1·5 - 3·6)	1·00	1·00	892, 762	1·1 (0·6 - 2·0)	1·00	1·00	888, 759
Increased	5·4 (3·5 - 8·4)	2·44 (1·28 - 4·68)	2·28 (1·18 - 4·38)	365, 290	0·5 (0·1 - 1·4)	0·42 (0·11 - 1·55)	0·36 (0·10 - 1·35)	369, 294
Decreased	5·3 (3·1 - 8·7)	2·36 (1·17 - 4·78)	1·93 (0·93 - 4·00)	282, 230	1·7 (0·7 - 3·9)	1·58 (0·54 - 4·62)	1·17 (0·39 - 3·51)	282, 229

**Symptoms of depression (PHQ-2) ******		**p=0·028**	**p=0·14**			**p=0·55**	**p=0·97**	
No	2·5 (1·7 - 3·6)	1·00	1·00	1054, 908	1·0 (0·5 - 1·8)	1·00	1·00	1054, 908
Yes	4·6 (3·1 - 6·9)	1·91 (1·07 - 3·41)	1·56 (0·87 - 2·79)	476, 367	1·3 (0·6 - 2·7)	1·34 (0·51 - 3·53)	1·02 (0·38 - 2·71)	477, 367
**Symptoms of anxiety (GAD-2) ******		**p=0·0026**	**p=0·022**			**p=0·082**	**p=0·25**	
No	2·5 (1·7 - 3·7)	1·00	1·00	986, 867	0·7 (0·4 - 1·5)	1·00	1·00	985, 866
Yes	5·7 (3·9 - 8·1)	2·37 (1·35 - 4·15)	1·96 (1·10 - 3·51)	555, 418	1·7 (0·9 - 3·2)	2·33 (0·90 - 6·04)	1·79 (0·67 - 4·77)	555, 418
**New sexual partners since lockdown [§]**		**p<0·0001**	**p<0·0001**			**p=0·019**	**p=0·055**	
None	2·1 (1·5 - 3·1)	1·00	1·00	1239, 1060	0·6 (0·3 - 1·2)	1·00	1·00	1238, 1059
At least one	23·9 (12·7 - 40·4)	14·4 (6·05 - 34·1)	10·5 (4·28 - 25·8)	44, 30 **	4·7 (1·0 - 19·3)	7·97 (1·40 - 45·3)	6·03 (0·96 - 38·0)	43, 29 **
**Condom-less sex with a new partner since lockdown [§]**		**p<0·0001**	**p<0·0001**			**p=0·049**	**p=0·11**	
None	2·3 (1·6 - 3·3)	1·00	1·00	1263, 1072	0·6 (0·3 - 1·2)	1·00	1·00	1261, 1070
At least one	[6·2 (13·0 - 45·7)	15·1 (5·85 - 39·0)	10·5 (3·94 - 28·2)	32, 22 **	4·8 (0·7 - 27·3)	8·32 (1·01 - 68·9)	6·22 (0·67 - 57·7)	31, 21 **
**Previous same-sex experience in their lifetime [‡]**		**p<0·0001**	**p<0·0001**			**p=0·0029**	**p=0·0072**	
No	2·9 (2·1 - 4·0)	1·00	1·00	1346, 1197	0·8 (0·5 - 1·5)	1·00	1·00	1346, 1196
Yes	12·1 (7·5 - 19·0)	4·62 (2·48 - 8·60)	4·21 (2·25 - 7·89)	202, 95	3·6 (1·7 - 7·7)	4·42 (1·66 - 11·79)	3·86 (1·44 - 10·3)	201, 94

CI=confidence intervals. OR=odds ratio. aOR=adjusted odds ratio. PHQ-2=Patient Health Questionnaire (2 item). GAD-2=Generalized anxiety disorder (2 item).

± Age-adjusted ORs, adjusting for age as a continuous variable
† Women aged 18-44 years who reported at least one sexual partner in the past year (i.e. sexually-active). Trans women are included in data for women. 12 and 13 sexually-active women aged 18-44 years responded "prefer not to say" to the questions about use and unsuccessful use of STI-related services, respectively. These individuals are excluded from the denominator.
* Unweighted denominator <30. Results not shown due to small denominator.
** Unweighted denominator <50. Results should be interpreted with caution due to small denominator.
*** White includes all those who identify as White English, Welsh, Scottish, Northern Irish, British, Irish, Gypsy or Irish Traveller, or from any other White background.
¶ Mixed ethnicity includes those who identify as White and Black African, White and Black Caribbean, White and Asian or any other mixed or multiple ethnic background.
¥ Asian includes those who identify as Indian, Pakistani, Bangladeshi, Chinese or from any other Asian background
¤ Black includes those who identify as African, Caribbean, or from any other Black background.
**** Participants were classified as having symptoms of depression or anxiety if they scored three or more on the patient health questionnaire two item (PHQ-2) or generalised anxiety disorder two item (GAD-2) scales
§ Includes both opposite-sex and same-sex partners
‡ Same-sex experience defined as oral/anal/vaginal sex

All percentages are weighted. These are row percentages which describe successful use/unsuccessful use of services within a specific subgroup.

Appendix G: Variations in reporting successful contraceptive service use and an unsuccessful attempt to use contraceptive services among sexually-experienced women aged 18-44 years (n=1715) in the four months following the start of a national lockdown in Britain (23/03/2020).

	Successful use				Unsuccessful attempt to use			
	% (95% CI)	OR (95% CI)	aOR (95% CI) ±	Denominator† (unweighted, weighted)	% (95% CI)	OR (95% CI)	aOR (95% CI) ±	Denominator† (unweighted, weighted)
**All sexually-experienced women aged 18-44 years**	14·8 (13·1 - 16·6)	··	··	1715, 1438	4·0 (3·1 - 5·1)	··	··	1715, 1438
**Age (years)**		**p<0·0001**				**p=0·58**		
18-24	22·3 (18·0 - 27·1)	2·96 (1·95 - 4·49)	··	370, 237	4·8 (3·0 - 7·8)	1·63 (0·77 - 3·46)	··	368, 236

25-29	21·0 (17·6 - 24·9)	2·75 (1·86 - 4·07)	··	550, 359	4·6 (3·0 - 6·9)	1·53 (0·76 - 3·10)	··	553, 361
30-34	12·3 (8·8 - 17·1)	1·46 (0·88 - 2·40)	··	287, 287	4·3 (2·4 - 7·6)	1·45 (0·64 - 3·26)	··	289, 290
35-44	8·8 (6·5 - 11·8)	1·00	··	508, 555	3·0 (1·8 - 5·1)	1·00	··	505, 551
**Location**		**p=0·11**	**p=0·10**			**p=0·038**	**p=0·039**	
Urban	15·3 (13·3 - 17·5)	1·00	1·00	1245, 1038	4·2 (3·2 - 5·5)	1·00	1·00	1245, 1038
Rural	11·1 (7·5 - 16·1)	0·69 (0·44 - 1·09)	0·68 (0·43 - 1·08)	222, 186	1·2 (0·4 - 3·8)	0·28 (0·09 - 0·93)	0·28 (0·09 - 0·94)	221, 185
**Ethnicity**		**p=0·56**	**p=0·73**			**p=0·16**	**p=0·19**	
White ***	14·5 (12·8 - 16·5)	1·00	1·00	1525, 1215	3·6 (2·8 - 4·8)	1·00	1·00	1526, 1216
Mixed/multiple/other ¶	29·7 (11·2 - 32·4)	1·45 (0·73 - 2·86)	1·36 (0·69 - 2·66)	59, 49	7·5 (2·7 - 19·0)	2·15 (0·72 - 6·46)	2·09 (0·69 - 6·27)	59, 49
Asian/ Asian British ¥	13·2 (7·0 - 23·6)	0·90 (0·44 - 1·84)	0·96 (0·45 - 2·03)	81, 111	3·4 (0·9 - 12·6)	0·94 (0·22 - 3·95)	0·97 (0·23 - 4·06)	81, 111
Black / Black British ¤	20·7 (9·6 - 38·9)	1·53 (0·62 - 3·79)	1·38 (0·56 - 3·43)	32, 45 **	10·3 (3·6 - 26·3)	3·06 (0·95 - 9·84)	2·91 (0·90 - 9·35)	32, 45 **
**Relationship status**		**p<0·0001**	**p=0·0023**			**p=0·44**	**p=0·50**	
Did not have partnered sex since lockdown	9·2 (6·4 - 12·9)	0·64 (0·41 - 0·98)	0·60 (0·39 - 0·94)	342, 284	2·3 (1·0 - 5·1)	0·53 (0·22 - 1·27)	0·52 (0·21 - 1·26)	341, 284
Partnered sex, not in a steady relationship	21·7 (16·1 - 28·7)	1·74 (1·15 - 2·65)	1·33 (0·86 - 2·03)	188, 143	5·4 (2·7 - 10·3)	1·25 (0·57 – 2·75)	1·08 (0·47 - 2·51)	188, 142
Partnered sex, in a steady, non-cohabiting relationship	26·3 (19·9 - 33·8)	2·24 (1·49 - 3·37)	1·61 (1·04 - 2·49)	174, 133	4·4 (2·2 - 8·6)	1·01 (0·46 - 2·24)	0·84 (0·37 - 1·87)	174, 133
Partnered sex, in a steady, cohabiting relationship	13·7 (11·6 - 16·2)	1·00	1·00	985, 857	4·3 (3·1 - 5·9)	1·00	1·00	985, 857
**Employment status**		**p=0.015**	**p=0.76**			**p=0.30**	**p=0.20**	

Employed	14·8 (12·9 - 16·9)	1·00	1·00	1315, 1118	4·4 (3·4 – 5·8)	1·00	1·00	1313, 1116
Unemployed	12·7 (7·8 - 19·9)	0·84 (0·48 - 1·47)	0·82 (0·47 - 1·46)	150, 118	1·4 (0·3 - 5·3)	0·30 (0·07 - 1·25)	0·30 (0·07 - 1·23)	151, 120
Full time parent, home maker	10·1 (5·7 - 17·3)	0·65 (0·34 - 1·24)	0·74 (0·38 - 1·43)	121, 118	2·5 (0·8 - 7·5)	0·55 (0·17 - 1·81)	0·59 (0·18 - 1·95)	122, 119
Student/Pupil	24·5 (17·5 - 33·2)	1·88 (1·19 - 2·96)	0·95 (0·59 - 1·54)	129, 83	3·5 (1·3 - 8·9)	0·77 (0·27 - 2·18)	0·53 (0·18 - 1·54)	129, 83
**Sexual identity - self-reported**		**p=0·0041**	**p=0·021**			**p=0·86**	**p=0·82**	
Heterosexual/straight	14·7 (13·0 - 16·7)	1·00	1·00	1472, 1366	4·0 (3·1 - 5·2)	1·00	1·00	1472, 1365
Gay or Lesbian	1·2 (0·2 - 7·9)	0·07 (0·01 - 0·50)	0·05 (0·01 - 0·40)	43, 16 **	4·6 (1·1 - 17·4)	1·16 (0·26 - 5·21)	1·07 (0·24 - 4·81)	43, 16 **
Bisexual	20·7 (15·2 - 27·5)	1·51 (1·01 - 2·26)	1·16 (0·77 - 1·75)	171, 38	3·1 (1·2 - 7·6)	0·77 (0·29 - 2·07)	0·68 (0·25 - 1·81)	170, 37
Other	··	··	··	18, 8 *	··	··	··	19, 9 *
**Social grade**		**p=0·30**	**p=0·29**			**p=0·69**	**p=0·71**	
AB Higher and intermediate managerial/administrative/professional occupations	17·0 (13·5 - 21·2)	1·00	1·00	433, 347	4·8 (2·9 - 7·7)	1·00	1·00	433, 346
C1 Supervisory, clerical and junior managerial/administrative/professional occupations/C2 Skilled manual occupations	14·6 (12·3 - 17·1)	0·83 (0·60 - 1·16)	0·86 (0·61 - 1·21)	897, 776	3·7 (2·6 - 5·2)	0·77 (0·41 - 1·44)	0·78 (0·42 - 1·47)	899, 778
D Semi-skilled and unskilled manual occupations/E On state benefit, unemployed and lowest grade occupations	12·9 (9·8 - 16·8)	0·73 (0·48 - 1·10)	0·71 (0·47 - 1·09)	385, 316	3·7 (2·0 - 6·6)	0·76 (0·35 - 1·68)	0·76 (0·35 - 1·69)	383, 313
**Education (3 categories)**		**p=0·21**	**p=0·21**			**p=0·73**	**p=0·74**	
Degree	14·4 (12·2 - 17·0)	1·00	1·00	961, 812	4·3 (3·1 - 6·0)	1·00	1·00	964, 815
Below degree	15·8 (13·2 - 18·8)	1·11 (0·84 - 1·48)	1·12 (0·84 - 1·49)	711, 591	3·5 (2·3 - 5·3)	0·81 (0·47 - 1·40)	0·81 (0·47 - 1·40)	709, 589

No qualifications	5·1 (1·2 - 18·7)	0·32 (0·07 - 1·38)	0·31 (0·07 - 1·39)	43, 35 **	3·3 (0·5 - 19·9)	0·75 (0·10 - 5·68)	0·76 (0·10 - 5·82)	42, 33 **
**Alcohol consumption since lockdown**		**p=0·43**	**p=0·37**			**p=0·89**	**p=0·89**	
No change	14·3 (12·1 - 16·7)	1·00	1·00	998, 855	3·8 (2·7 - 5·3)	1·00	1·00	995, 853
Increased	17·0 (13·4 - 21·2)	1·23 (0·88 - 1·72)	1·16 (0·82 - 1·63)	393, 312	4·4 (2·7 - 7·0)	1·15 (0·62 - 2·11)	1·11 (0·61 - 2·04)	397, 316
Decreased	13·9 (10·3 - 18·5)	0·97 (0·66 - 1·43)	0·84 (0·56 - 1·25)	315, 261	3·7 (1·9 - 7·2)	0·97 (0·44 - 2·13)	0·90 (0·40 - 2·04)	315, 260
**Symptoms of depression (PHQ-2) ******		**p=0·010**	**p=0·066**			**p=0·25**	**p=0·35**	
No	13·3 (11·3 - 15·5)	1·00	1·00	1160, 1005	3·4 (2·5 - 4·8)	1·00	1·00	1161, 1007
Yes	18·3 (15·1 - 22·1)	1·47 (1·09 - 1·97)	1·32 (0·98 - 1·79)	537, 415	4·7 (3·1 - 7·1)	1·38 (0·79 - 2·41)	1·31 (0·75 - 2·30)	538, 415
**Symptoms of anxiety (GAD-2) ******		**p=0·075**	**p=0·45**			**p=0·0028**	**p=0·0063**	
No	13·6 (11·6 - 15·8)	1·00	1·00	1097, 970	2·9 (2·0 - 4·2)	1·00	1·00	1097, 970
Yes	17·0 (14·0 - 20·4)	1·30 (0·97 - 1·74)	1·12 (0·83 - 1·52)	610, 461	6·3 (4·4 - 8·9)	2·24 (1·32 - 3·80)	2·12 (1·24 - 3·63)	610, 460

CI=confidence intervals. OR=odds ratio. aOR=adjusted odds ratio. PHQ-2=Patient Health Questionnaire (2 item). GAD-2=Generalized anxiety disorder (2 item).

± Age-adjusted ORs, adjusting for age as a continuous variable
† Women, including trans women, aged 18-44 years who reported at least one sexual partner in their lifetime (i.e. sexually-experienced). 13 sexually-experienced women aged 18-44 years responded “prefer not to say” to the questions about use and unsuccessful use of contraceptive services. These individuals are excluded from the denominator.
* Unweighted denominator <30. Results not shown due to small denominator.
** Unweighted denominator <50. Results should be interpreted with caution due to small denominator.
*** White includes all those who identify as White English, Welsh, Scottish, Northern Irish, British, Irish, Gypsy or Irish Traveller, or from any other White background.
¶ Mixed ethnicity includes those who identify as White and Black African, White and Black Caribbean, White and Asian or any other mixed or multiple ethnic background.
¥ Asian includes those who identify as Indian, Pakistani, Bangladeshi, Chinese or from any other Asian background
¤ Black includes those who identify as African, Caribbean, or from any other Black background.

**** Participants were classified as having symptoms of depression or anxiety if they scored three or more on the patient health questionnaire two item (PHQ-2) or generalised anxiety disorder two item (GAD-2) scales

All percentages are weighted. These are row percentages which describe successful use/unsuccessful use of services within a specific subgroup.
